# A novel long noncoding RNA linc00460 up-regulated by CBP/P300 promotes carcinogenesis in esophageal squamous cell carcinoma

**DOI:** 10.1042/BSR20171019

**Published:** 2017-10-17

**Authors:** Yan Liang, Yuanyuan Wu, Xuedan Chen, Shixin Zhang, Kai Wang, Xingying Guan, Kang Yang, Juan Li, Yun Bai

**Affiliations:** 1 Department of Medical Genetics, College of Basic Medical Science, Third Military Medical University, Gaotanyan Street, Shapingba District, Chongqing, China; 2 Department of Cardiothoracic Surgery, Southwest Hospital, Third Military Medical University, Gaotanyan Street, Shapingba District, Chongqing, China

**Keywords:** apoptosis, CBP/P300, cell proliferation, ESCC, long intergenic non-protein coding RNA 460

## Abstract

Esophageal cancer is one of the leading causes of cancer-related mortality because of poor prognosis. Long noncoding RNAs (lncRNAs) have been gradually demonstrated to play critical roles in cancer development. We identified a novel long noncoding RNA named linc00460 by microarray analysis using esophageal squamous cell carcinoma (ESCC) clinical samples, which has not been studied before. Our research indicated that linc00460 was overexpressed in the majority of tumor tissues and ESCC cell lines. Linc00460 expression was positively correlated with ESCC TNM stage, lymph node metastasis, and predicted poor prognosis. *In vitro* experiments showed that linc00460 depletion suppressed ESCC cell growth through regulating cell proliferation and cell cycle; in additional, linc00460 depletion accelerated ESCC cell apoptosis. We further revealed that linc00460 overexpression was manipulated by transcriptional co-activator CBP/P300 through histone acetylation. Given the high expression and important biological functions of linc00460, we suggest that linc00460 works as an oncogene and might be a valuable prognostic biomarker for ESCC diagnosis and treatment.

## Introduction

Esophageal cancer is the 8th most commonly diagnosed cancer type and the 6th leading cause of cancer-related death, with an estimated 456,000 new cases each year worldwide [1,2]. The esophageal cancer morbidity of men is 3-fold higher than women, and mostly occurred in rural areas [3]. Alcohol addiction and tobacco abuse are proved to be risk factors for esophageal cancer [4] with other carcinogens, such as HPV infection [5]. Esophageal squamous cell carcinoma (ESCC) is the major histological type versus esophageal adenocarcinoma, accounting for 80% of esophageal cancer cases in South-Eastern and Central Asia [6]. As >50% of esophageal cancers are unresectable or with local invasion and organ metastasis at the time of diagnosis, only 15–25% of the patients can survive over 5 years despite of the improvements of surgery or other comprehensive treatments [7]. Therefore, investigating the mechanism underlying esophageal cancer initiation and development is critical for exploiting new diagnosis markers and therapeutic targets [8].

Long noncoding RNAs (lncRNAs) are defined as novel RNAs >200 nt in length, occupied at least 80% of human genome with no protein coding potential [9]. These ncRNAs are usually transcribed by RNA polymerase II, also spliced and mostly polyadenylated, analogues to protein coding genes [10]. Researchers have recognized many functional lncRNAs participating in tumorigenesis, due to their irregular expression and specific expression patterns in various tumor types [11–13]. These lncRNAs have been demonstrated to modulate cancer cell behaviors including cancer progression, cell metastasis, and drug resistance. Some of the first identified lncRNAs such as HOX transcript antisense intergenic RNA (HOTAIR) [14], MALAT1 [15], and H19 [16] are highly expressed in multiple tumor tissues and play regulatory roles in chromatin remodeling through histone modification, DNA methylation, or function as competing endogenous RNAs through interacting with microRNAs [17]. Previous studies have identified that some lncRNAs expressed in ESCC disorderly, affecting cancer development and prognosis [18]. However, the delicate molecular basis of lncRNA function relies on deeper research and advances in scientific technology.

In the present study, we identified many differentially expressed lncRNAs by microarray analysis using five paired ESCC clinical tissues. Microarray results indicated that a newly identified lncRNA named long intergenic nonprotein coding RNA 460, abbreviated to linc00460, had a relatively great alteration between cancer tissues and normal tissues. As a novel identified lncRNA, the expression and function of linc00460 in ESCC were unknown. Our research indicated that linc00460 might function as a novel oncogene and might be a valuable prognostic biomarker for ESCC diagnosis and treatment.

## Materials and methods

### Patients and clinical tissues

ESCC tissues and corresponding adjacent normal tissues were collected from patients underwent esophagectomy in Southwest Hospital (the Third Military Medical University, Chongqing China) between 2006 and 2014. Our research project was approved by constituted Ethics Committee of the university and it conformed to the provisions of the Declaration of Helsinki. All patients enrolled were informed and consent. None of them received chemo- or radiotherapy or other preoperative treatments before surgery. The clinical tissues were stored at liquid nitrogen immediately after surgery. Patients were staged according to the American Joint Committee on Cancer (AJCC) by at least two pathologists. Our research observed the Declaration of Helsinki and was approved by the Human Ethics Committee of the Third Military Medical University.

### Microarray screening and bioinformatics analysis

The microarray profiling was performed using five paired ESCC tissues and normal tissues, the clinical tissues were obtained from five ESCC male patients who were clinically diagnosed and pathologically confirmed, and none of them received chemo- or radiotherapy or other preoperative treatments before surgery. RNA extraction and sequential microarray hybridization were conducted by Kangchen Company (Shanghai, China), the detected human genome transcripts were from the Human lncRNA microarray v2.0 (8×60 K, arraystar, U.S.A.). Hierarchical cluster analysis was performed using Cluster software to obtain differential expressed lncRNAs and mRNAs. The results are available in Gene Expression Omnibus (GEO) with the serial number GSE89102.

### Cell culture

The human ESCC cell lines EC109, KYSE150, and KYSE450 were purchased from the Cell Bank of the Chinese Academy of Science (Shanghai, China), the human normal esophageal epithelial cell line Het-1A was purchased from American Type Culture Collection (Maryland, U.S.A.). The other cancer cell lines used in the present study were preserved by our laboratory for years. EC109, KYSE150, KYSE450, and Het-1A cell lines were verified by STR genotype method at Key Laboratory of Birth Defects and Reproductive Health (Chongqing, China) (Supplementary Figure S1). All cells were cultured in RPMI-1640 medium (Hyclone, U.S.A.), containing 10% newborn bovine serum; Het-1A was cultured in BEGM medium from Lonza/Clonetics Corporation; all cells were maintained in a humidified incubator at 37°C containing 5% CO_2_.

### RNA extraction and qRT-PCR assay

Total RNA was isolated from either clinical tissues or cultured cells using the Trizol reagent (Takara, Japan) according to the manufacturer’s instructions. The RNA concentration and quality were measured by NanoDrop ND-1000 spectrophotometer. First strand cDNA was synthesized from 200 ng of total RNA using the PrimeScript RT reagent Kit (Takara, Japan). SYBR Premix Ex Taq (Takara, Japan) was used for Quantitative real-time PCR assay on the CFX Connect Real-Time System (Bio-Rad, U.S.A.). The primers used for qPCR were listed in Supplementary Table S1. The relative expression of linc00460 was calculated from the formula 2^−ΔΔ*C*t^ and normalized with GAPDH.

### SiRNA transfection

Small interfering RNAs (siRNAs) were designed and synthesized by Shanghai GenePharma (Shanghai, China). The siRNA sequences used in the present study were listed in Supplementary Table S2*.* The cells were seeded and cultured in six-well plate with density of 3 × 10^5^/well overnight. Then, cells were transfected with siRNAs or negative control at a final concentration of 50 nM using Lipofectamin 2000 reagent (Invitrogen, Carlsbad, U.S.A.) according to the manufacturer’s instruction.

### Cell growth and proliferation assay

ESCC cells transfected with siRNAs or negative control were seeded in 96-well plate (5 × 10^3^/ well). Cell growth assay was performed using Cell Counting Kit-8 (CCK-8) (Dojindo Laboratory, Japan) every 24 h according to the protocol. The number of viable cells was quantified by the absorbance of 450 nm using the microplate reader.

After transfection for 48 h, cell proliferation assay was performed using Cell-Light^TM^ Edu Apollo567 In Vitro Kit (Ribobio, China) with fluorescence microscope according to the protocol. The cell proliferation rate was calculated according to Edu incorporation rate. Each experiment group had three replicates.

### Cell cycle and apoptosis analysis

After transfected with siRNA for 48 h, cells were harvested by trypsin, and then cells were fixed with 70% ethanol at 4°C overnight. Then fixed cells were incubated with RNase A for 30 min to completely degrade RNA, and then stained with propidium oxide for another 30 min in dark place using Cell Cycle Analysis Kit (Beyotime Biotechnology, China). The managed cells were detected by flow cytometer FACSCalibur (BD Bioscience, U.S.A.) and analyzed with Flowjo software.

For apoptosis analysis, transfected cells were collected by trypsin 48 h after transfection, and then stained with Annexin V-FITC and PI using Cell apoptosis Analysis Kit (Beyotime Biotechnology, China). Stained cells were detected by flow cytometer FACSCalibur (BD Bioscience, U.S.A.) and analyzed with Flowjo software.

### Nucleus and cytoplasm isolation assay

Nucleus and cytoplasm isolation assay was performed using Nuclei Isolation Kit: Nuclei Ez Prep (Sigma, U.S.A.) according to manufacturer‘s instruction. Then we used Trizol reagent to extract RNA from isolated nucleus and cytoplasm fraction. Reverse transcription and PCR reaction were done as described before. GAPDH and U6 were used as important criteria of isolation quality. The primers of GAPDH and linc00460 used for PCR were same with qPCR primers described before; the primers of U6 were purchased from GeneCopoeia^TM^ (#HmiRQP9001, China).

### Chromatin immunoprecipitation assay

Chromatin immunoprecipitation (ChIP) was performed using the SimpleChIP® Enzymatic Chromatin IP Kit (#9003, CST, U.S.A.) according to the manufacturer’s instruction. Cells were harvest at 80–90% confluence after siRNA transfection. Briefly, cells were cross-linked with 1% formaldehyde for 15 min, and then terminated with glycine before scrapped from culture dishes. Afterwards, the collected cells were digested with nuclease to break the cross-linked chromatin to 100–200 bp length. Then appropriate amount of chromatin was immunoprecipitated using anti-CBP (ab2832, Abcam, U.S.A.), anti-P300 (ab14984, Abcam, U.S.A), anti-histone H3A (acetyl K27) (ab4729, Abcam, U.S.A.), and anti-histone H3A (acetyl K18) (Cat.#17-10111, Millipore, U.S.A.); goat anti-Rabbit IgG and goat anti-Mouse IgG were used as negative control respectively. The immunoprecipitation reaction was performed overnight at 4°C with rotation. The next day, the precipitated chromatin were washed and eluted from the antibody/protein G magnetic beads, and then DNA purification was performed and analyzed by qPCR with specific primers listed in Table 1.

**Table 1:** Correlation between linc00460 expression and ESCC clinical parameters

		Low-expression	High-expression	*P* value^a^
Clinical parameters		*N*=33	*N*=32	
Age	<60 years	23	19	
	>60 years	10	13	0.384
Gender	Male	24	27	
	Female	9	5	0.253
Lymph node metastasis	Negative	24	15	
	Positive	9	17	0.033*
TNM stage	I/II	28	17	
	III/IV	5	15	0.006**
Histological differentiation	Well/moderate	31	27	
	Poor	2	5	0.258
Smoking	Yes	21	24	
	No	12	8	0.321
Drinking	Yes	19	25	
	No	14	7	0.077

^a^Chi-squared test results

*P<0.05

**P<0.01

### Statistical analysis

Experimental data are presented as mean ± SE from three independent experiments in triplicate. Statistical analysis was performed using the SPSS software package version 16.0. For comparison, paired or independent Student’s *t*-test, Chi-square test, or one-way ANOVA were chosen as appropriate. Kaplan–Meier method and log-rank test were used to delineated survive curve. All *P* values were two sided, and a *P*<0.05 was considered significant.

## Results

### Linc00460 is a novel long noncoding RNA with potential function in ESCC

In order to identify aberrantly expressed lncRNAs in human esophageal squamous cell carcinoma, we performed microarray analysis using five paired ESCC tissues and adjacent nontumor tissues. The results revealed that 2939 (12.4%) lncRNA transcripts were up-regulated (fold change>2, *P*<0.05), and 3517 (14.64%) lncRNA transcripts were down-regulated (fold change<0.5, *P*<0.05) in ESCC tissues compared with normal tissues. The microarray results were validated by qRT-PCR and performed high repeatability (Supplementary Figure S2). Of all differentially expressed lncRNAs, linc00460 was one of the mostly up-regulated lncRNAs in tumor tissues (fold change = 41.9, *P*=0.006), which aroused our attention.

**Figure 1:**
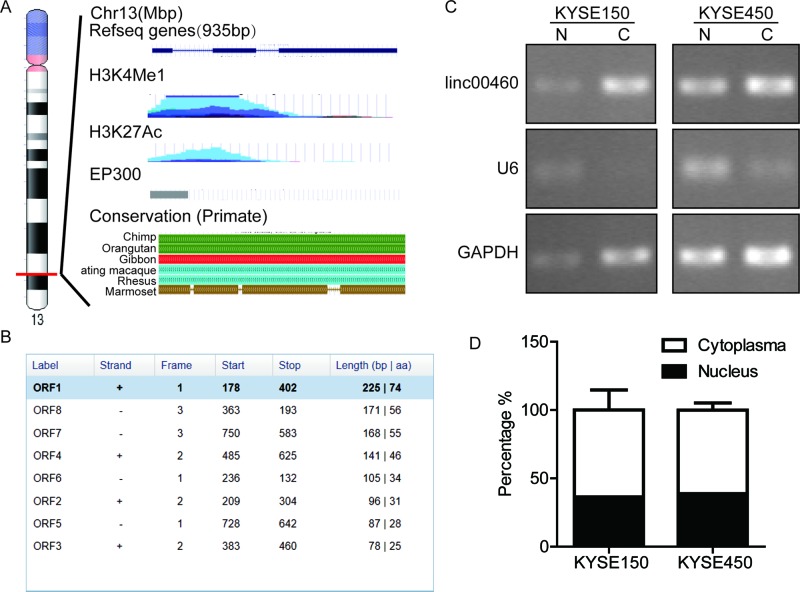
Linc00460 is a novel long noncoding RNA with potential function in ESCC (**A**) The genomic construction of linc00460 coding region according to UCSC dataset (February 2009, GRCH37/hg19). (**B**) Results of ORFfinder software to predict protein sequence of linc00460. (**C**) Linc00460 subcellular localization analyzed by nucleus and cytoplasm isolation assay. Electrophoresis analysis of PCR products synthesized from nucleus and cytoplasm fraction of KYSE150 and KYSE450. N represents for nucleus, C represents for cytoplasm. (**D**) Percentage bar chart represents for linc00460 distribution between nucleus and cytoplasm in KYSE150 and KYSE450. Data were presented as mean ± SE from three independent experiments.

According to RefSeq database, linc00460 is located at chromosome 13 in human genome, the transcript length is 935 bp (NR_034119, GenBank), consisting of three exons (Figure 1A). Using ORFfinder from NCBI we failed to predict a protein sequence longer than 80 amino acids (Figure 1B), strongly suggesting that linc00460 lacked protein coding capacity. Bioinformatic analysis indicated that linc00460 was transcribed from a gene desert region, suggesting that linc00460 belonged to long intergenic noncoding RNA (lincRNA). The conservation of linc00460 genomic region among primate genomes indicated its importance in evolution (Figure 1A). In addition, linc00460 had been detected to be dysregulated in a multitude of physiology and pathology processes according to GEO Profiles database. For instance, linc00460 expression was significantly lower in Neural tube defect patient than normal (GDS2470) (Supplementary Figure S3A); linc00460 expression was decreased when knockdown LSD1 in neuroblastoma cell lines (GDS5281) (Supplementary Figure S3B); MTX-sensitive HT29 colon adenocarcinoma cell line presented higher linc00460 expression than MTX-resistant HT29 colon adenocarcinoma cell line (GDS3330) (Supplementary Figure S3C). These suggested that linc00460 might function in organ development and tumorigenesis. Thus, we considered linc00460 might function in ESCC carcinogenesis and further detected the expression and function in ESCC tissues.

To determine the subcellular localization of linc00460 in ESCC cells, we also performed nucleus and cytoplasm isolation assay. The results in KYSE150 and KYSE450 cells showed that linc00460 was located both in nucleus and cytoplasm, mainly in cytoplasm (Figure 1C and D).

### Linc00460 is overexpressed in ESCC tissues and correlated with ESCC clinical characteristics

**Figure 2:**
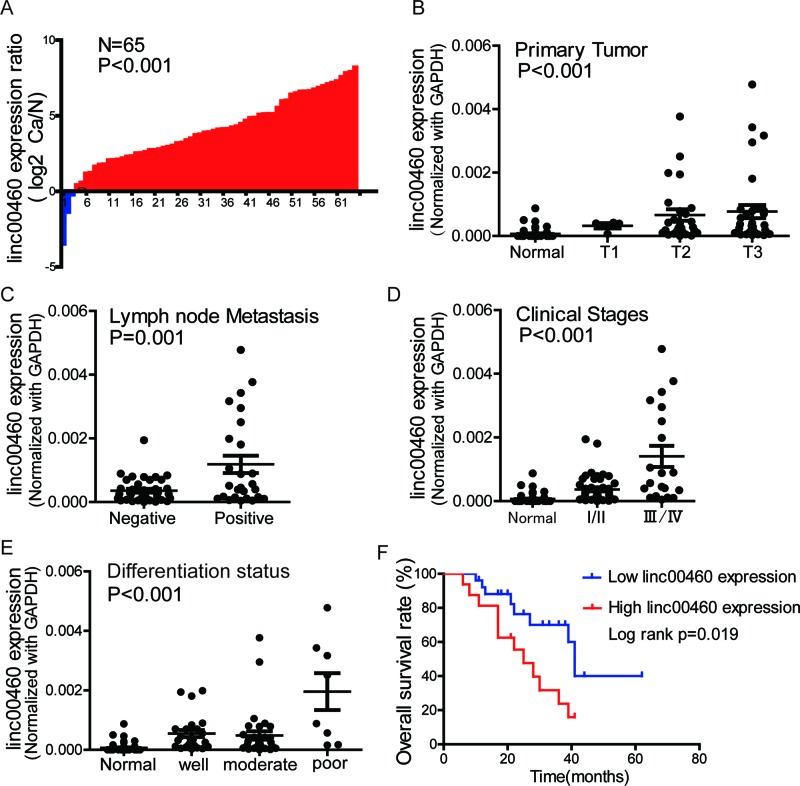
Linc00460 expression is higher in ESCC tissues and correlated with ESCC clinical characteristics (**A**) Fold change of linc00460 expression of ESCC tissues to adjacent normal tissues normalized by log2. The red column indicates linc00460 expression was higher in ESCC tissues, while the blue column indicates the opposite. (**B**) Linc00460 expression was positively correlated with ESCC primary tumor invasion depth. The statistical significance was calculated by independent-samples Student’s *t*-test. (**C**) ESCC patients with lymph node metastasis had higher linc00460 expression. The statistical significance was calculated by independent-samples Student’s *t*-test. (**D**) The expression of linc00460 was significantly up-regulated in higher TNM stage of ESCC tissues. The statistical significance was calculated by one-way ANOVA analysis. (**E**) The worse differentiation status of ESCC was correlated with high linc00460 expression. The statistical significance was calculated by one-way ANOVA analysis. (**F**) Kaplan–Meier analysis of overall survive rate of 42 patients indicated that higher linc00460 expression exhibited poorer overall survive. *P* value was calculated by log-rank test.

To further confirm linc00460 expression pattern in ESCC, we performed qRT-PCR using ESCC clinical tissues and normal tissues. We found that linc00460 expression was higher in 95.4% (62/65) of the ESCC tissues compared with adjacent normal tissues (Figure 2A). Linc00460 expression was positively correlated with ESCC primary tumor invasion depth (Figure 2B). Patients with lymph node metastasis and later TNM stage had higher linc00460 expression level (Figure 2C and D). The worse differentiation status of ESCC was correlated with high linc00460 expression (Figure 2E). The positive rate of linc00460 overexpression in ESCC was remarkably, indicating that it could be used as a biomarker for ESCC molecular diagnosis clinically.

To further explore the relationship between linc00460 expression and ESCC clinical characteristics, we divided the 65 ESCC patients into two groups according to linc00460 expression: the linc00460 high-expression group (*n*=32, fold change > average) and the linc00460 low-expression group (*n*=33, fold change < average). Statistical analysis suggested that linc00460 high-expression group presented more lymph node metastasis and later TNM stage than linc00460 low-expression group. However, there was no difference of histological differentiation, smoking, and drinking status between these two groups (Table 1). To investigate the relationship between linc00460 expression and ESCC prognosis, we delineated survive curve using Kaplan–Meier method. The results showed that linc00460 expression was in inverse proportion to ESCC survive rate (Figure 2F). All the above results indicated that up-regulation of linc00460 was a common effect in the carcinogenesis of ESCC and predicted poor prognosis.

### Linc00460 promotes ESCC cell growth and apoptosis *in vitro*

**Figure 3:**
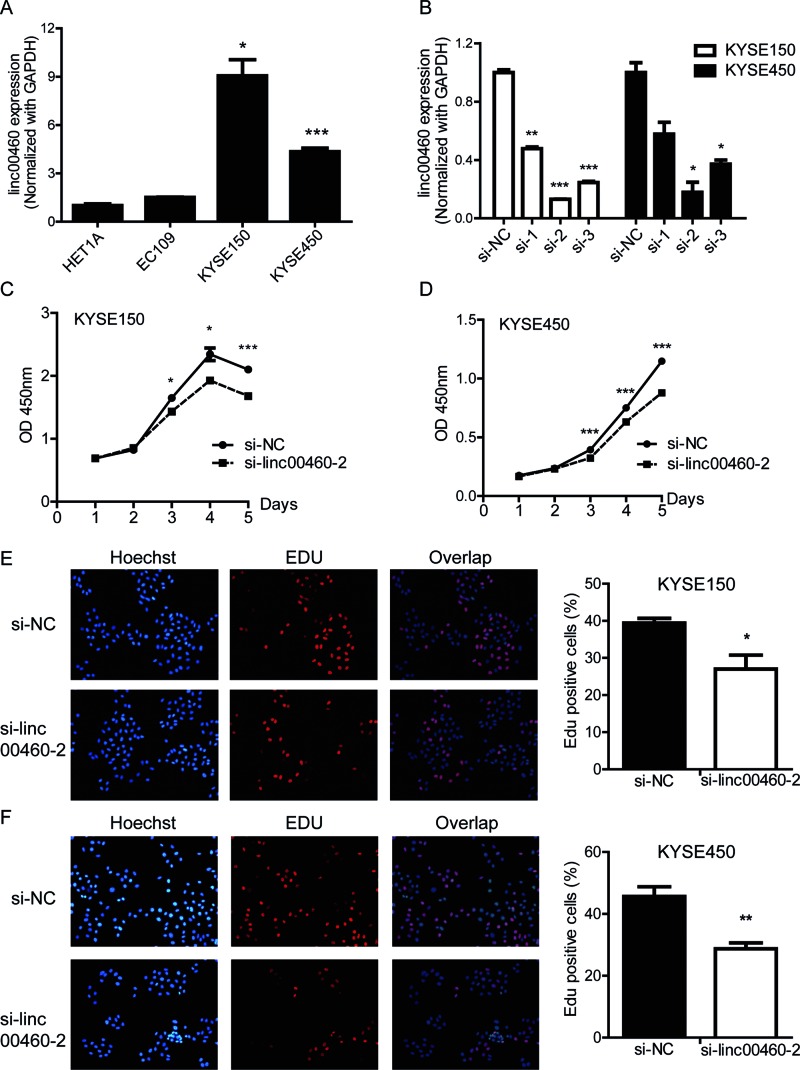
Linc00460 promotes ESCC cells growth *in vitro* (**A**) Linc00460 expression in three ESCC cell lines (EC109, KYSE150, and KYSE450) and a normal esophageal epithelial cell line Het-1A. Data were presented as expression fold-change relative to Het-1A. (**B**) Linc00460 expression of KYSE150 and KYSE450 after transfection of three different siRNAs targeting linc00460 and negative control. Si2 was used in the present study. (**C** and **D**) CCK-8 assay indicated that knockdown of linc00460 expression decreased cell growth in KYSE150 and KYSE450. (**E** and **F**) EDU incorporation assay indicated that linc00460 knockdown decreased cell proliferation in KYSE150 and KYSE450. Data were presented as mean ± SE from three independent experiments in triplicate; *P<0.05, **P<0.01, and ***P<0.001.

In order to explicit the meaning of linc00460 overexpression, we next investigated its biological function in ESCC. First, we measured linc00460 expression in three human ESCC cell lines (EC109, KYSE150, and KYSE450) and one human normal esophageal epithelial cell line (Het-1A), we found that the linc00460 expression in KYSE150 and KYSE450 was significantly higher than that in EC109 and Het-1A, especially the expression in KYSE150 (Figure 3A). To our knowledge, EC109 and KYSE450 were established from ESCC surgical specimen with well-differentiated histology, whereas KYSE150 was established from poor differentiated ESCC carcinoma [19]. So, the particularly high expression of linc00460 in KYSE150 was in accordance with the expression pattern in ESCC tissues. Additionally, we measured linc00460 expression in multiple cancer cell lines, the results indicated that linc00460 was overexpressed in many digestive system cancers (Supplementary Figure S4).

To investigate the biological functions of linc00460 on ESCC *in vitro*, three different siRNAs were used to knockdown linc00460 expression in KYSE150 and KYSE450 that had higher level of linc00460. The interference efficiency was tested 48 h after transfection, as shown in Figure 3B, the si2 presented the best interference efficiency, which was used in the following experiments. The CCK-8 assay showed that the vital cell number of KYSE150 and KYSE450 transfected with linc00460 siRNA was less than the control group (Figure 3C and D), indicated that linc00460 promoted ESCC cell growth. Since both cell proliferation and apoptosis can contribute to the effect on cell growth, we further studied the function of linc00460 on the two aspects.

**Figure 4:**
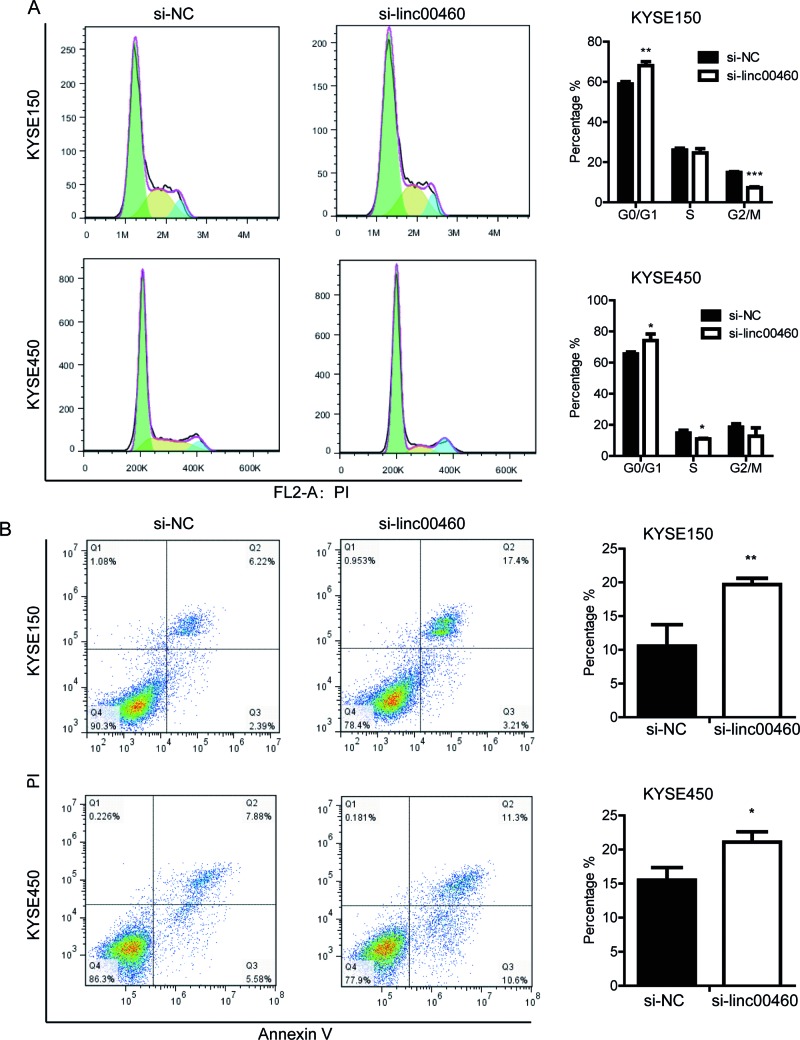
Linc00460 knockdown affects ESCC cell cycle and cell apoptosis (**A**) Representative images of cell cycle distribution of siRNA transfected KYSE150 and KYSE450, the bar diagram indicated the percentage of cells distributed in G0/G1, S, and G2/M phases. (**B**) Representative images of cell apoptosis of siRNA transfected KYSE150 and KYSE450, the bar diagram indicated the overall apoptosis rates. Data were presented as mean ± SE from three independent experiments in triplicate; *P<0.05, **P<0.01, and ***P<0.001.

EDU proliferation assay showed that the incorporation rate of KYSE150 and KYSE450 decreased after transfected with linc00460 siRNA (Figure 3E and F), demonstrating that depletion of linc00460 damaged ESCC cell proliferation. Then we performed flow cytometer experiment to test cell cycle and cell apoptosis. The results showed that in KYSE150, linc00460 depletion resulted in the increase in G0/G1-phase distribution and decrease in G2/M-phase distribution (Figure 4A); however, in KYSE450, linc00460 depletion only resulted in decrease of S-phase distribution (Figure 4A). Moreover, cell apoptosis rates in KYSE150 and KYSE450 were increased after linc00460 siRNA transfection (Figure 4B). These results indicated that cell growth induced by linc00460 was probably due to both cell proliferation promotion and apoptosis inhibition.

### CBP/P300 binding to linc00460 promoter activates linc00460 transcription through histone acetylation

**Figure 5:**
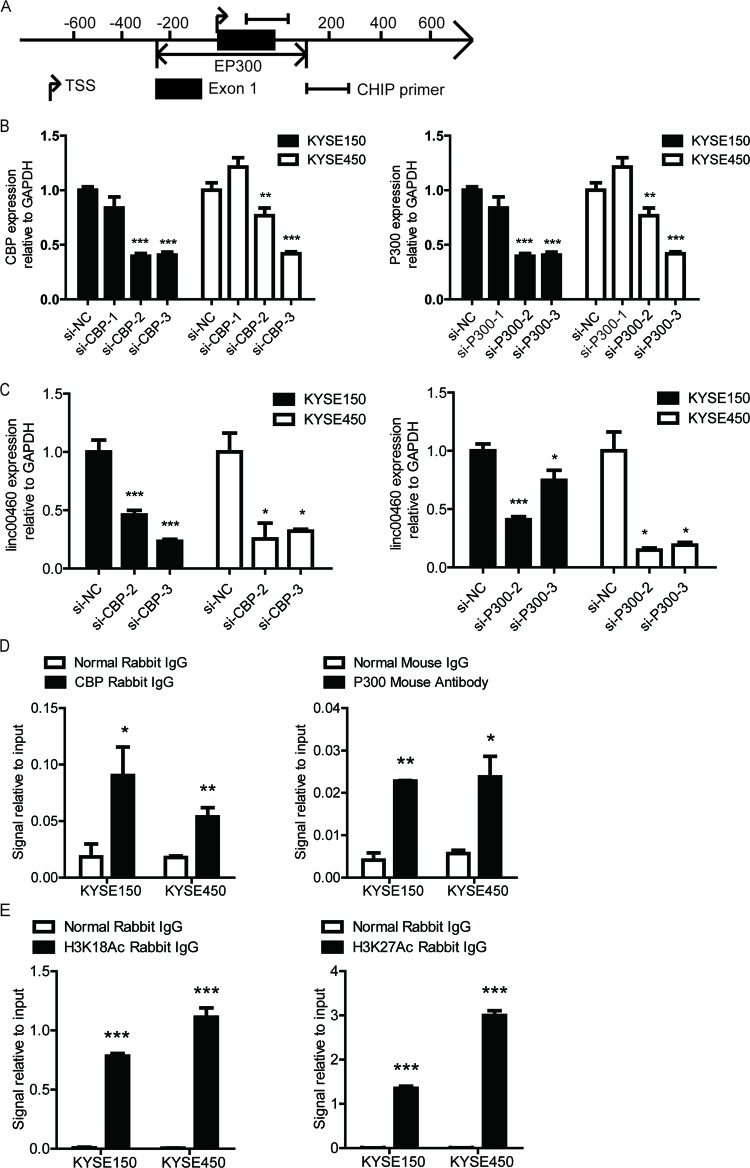
CBP/P300 binding to linc00460 promoter activates linc00460 transcription through histone acetylation (**A**) Schematic diagram of linc00460 promoter. TSS represents transcription start site, represents P300 binding region. (**B**) CBP or P300 expression after transfection with siRNAs and negative control in KYSE150 and KYSE450 detected with qRT-PCR assay. (**C**) Linc00460 expression after transfection with CBP or P300 siRNAs and negative control in KYSE150 and KYSE450 detected with qRT-PCR assay. (**D**) CHIP-qPCR analysis of KYSE150 and KYSE450 using CBP and P300 antibody. (**E**) CHIP-qPCR analysis of KYSE150 and KYSE450 using H3K18Ac and H3K27Ac antibody; *P<0.05, **P<0.01, and ***P<0.001.

Next, we asked why linc00460 was up-regulated in ESCC tissues. Bioinformatics analysis revealed that the promoter region of linc00460 was enriched of many histone modification signals, such as H3K4Me1, H3K4Me3, and H3K27Ac signals (Figure 1A); in addition, Transcription Factor ChIP-seq experiments performed by ENCODE project showed that many transcription factors and transcription co-activators could bind to linc00460 promoter, such as GATA2, CEBPB, P300, Fos, Jun etc., indicating that the linc00460 gene region was capable of transcription and could be regulated by chromatin modification (Figure 1A). CBP and P300 are closely related transcriptional co-activators and acetyltransferase enzymes in humans, which have been reported to activate gene expression through binding specific transcription factors to transcript machinery and chromatin modulation [20]. Considering that the promoter of linc00460 contains P300 binding signal (Figure 5A), we hypothesized that CBP and P300 might activate linc00460 transcription as co-activators by modifying histone acetylation in ESCC.

In order to verify our hypothesis, first, we knockdown CBP and P300 expression using siRNAs targeting three different sites respectively, the siRNAs with ideal interference efficacy were chosen for next experiments. The qRT-PCR results showed that both CBP and P300 depletion reduced linc00460 expression in KYSE150 and KYSE450 (Figure 5B and C). Then, we employed ChIP-qPCR assay to detect whether CBP and P300 bind to linc00460 promoter. The results showed that both individual CBP and P300 proteins could bind to linc00460 promoter (Figure 5D); meanwhile, we also detected high acetyl-Histone H3 (Lys18 and Lys27) enrichment signal in linc00460 promoter (Figure 5E). Therefore, we concluded that CBP/P300 binding to linc00460 promoter activates linc00460 transcription through histone H3 acetylation. The acetyl histone loosens the chromatin structure, this puffy conformation allows easier access for transcription machinery.

## Disscusion

LncRNAs exists prevalently in human genome, characterization of lncRNAs function in cancer has been of ongoing interest [21]. In recent years, accumulating evidence prove that lncRNA dysregulation play important roles in ESCC carcinogenesis, cancer development, metastasis, and patient outcome. Our laboratory discovered that lncRNA MALAT1 and H19 expression were significantly higher in ESCC and were correlated with tumor TNM stage, lymph node metastasis [22,23], which was with accordance to other researches [24,25]. Besides, lncRNAs such as HOTAIR [26–28], antisense noncoding RNA in the INK4 locus (ANRIL) [29–31], and colon cancer associated transcript-1 (CCAT1) [32–34] have been reported to be up-regulated in at least three types of digestive system cancers (DSCs) including ESCC; in additional, there are many other lncRNAs investigated in ESCC, such as linc-POU3F3 [35], AFAP1-AS1 [36], HNF1A-AS1 [37], HOTTIP [38] etc. Recently, scientists found that the circulating expression level of linc-POU3F3 in plasma showed reliable potential for ESCC diagnosis [39]; on the other hand, HOTAIR was the most commonly identified negative biomarker for ESCC prognosis [40]; moreover, Li et al. [41] established a three-lncRNA signature (ENST00000435885.1, XLOC_013014, and ENST00000547963.1) as a new independent biomarker for ESCC prognosis. However, the clinical application of lncRNAs as therapeutic targets in ESCC hasn’t been conducted.

In the present study, we identified a functional-unknown lncRNA named linc00460 through microarray analysis in ESCC tissues. We found that linc00460 was up-regulated in ESCC and was correlated with ESCC aggressiveness. Linc00460 exerted its oncogenic roles through regulating ESCC cell proliferation, cell cycle, and apoptosis. Our research revealed a remarkably high positive rate of linc00460 overexpression in ESCC clinical tissues, indicating that linc00460 can be a potential biomarker for ESCC molecular diagnosis. In view of the association of linc00460 expression with ESCC clinical characteristics, linc00460 could be a potential index for monitoring ESCC development and prognosis, which needs further study and analysis in a larger number of clinical samples.

LncRNA overexpression is widely reported in different species and organs. The mechanisms underline could be genome variation [22,42], transcriptional activation by classical oncogenes [43], transcription factors regulation, chromatin modification epigenetically [36], and microRNAs [25]. Among these, CBP and P300 were reported to be important regulators. CBP (also called CREB-binding protein, CREBBP, or KAT3A) and P300 (also called EP300 or KAT3B) were first identified and investigated as members of E1A interacting proteins [44]. They have significant sequence homology and many overlapping functions, thus the two proteins are now referred as CBP/P300 [44,45]. CBP/P300 was traditionally recognized to be involved in the transcriptional activation of many protein coding genes [44,46,47], recently, CBP/P300 was reported to regulate lncRNA expression. It has been reported that HOTAIR was overexpressed in breast cancer partly because of the binding of the CBP/P300 to the promoter of HOTAIR [48]; also, it has been reported that CREB unregulated the expression of lncRNA HULC through binding to the core promoter of this lncRNA [49], whereas CBP/P300 can interact with CREB directly.

In the present study, we discovered that CBP/P300 binds to linc00460 promoter; meanwhile, we detected acetylation toward H3K18 and H3K27, which gives an epigenetic tag for transcriptional activation. This phenomenon suggested that the binding of CBP/P300 to linc00460 promoter modulates the closed, silenced chromatin to open, permissive chromatin. This chromatin architecture remolding facilitates transcription machinery access and activates transcription consequently. To our knowledge, CBP/P300 can be downstream effectors of many signaling pathways, such as NF-κB signaling pathway, Notch signaling pathway, hypoxia, and DNA damage [45,50–52]. Thus, we may draw a picture that the above biological pathways alteration result in abnormal gene expression such as linc00460 through CBP/P300 function, and finally causes tumor formation and development.

In conclusion, we identified a novel lncRNA named linc00460, which acted as oncogene in ESCC; CBP/P300 up-regulated linc00460 expression through binding to linc00460 promoter and modulating chromatin architecture; linc00460 could be a candidate biomarker for ESCC diagnosis and treatment.

## Supporting information

**Figure S1:** The results of cell line STR genotype. A, B, C, D represents for EC109, KYSE150, KYSE450 and Het-1A respectively.

**Figure S2:** qRT-PCR confirmed the PCR results of microarray. (A, B) LncRNA BC062758 and AC098973.2 were upregulated in ESCC tissues compared with normal tissues detected by microarray analysis and qRT-PCR; (C. D) LncRNA CCAT1 and LINC00443 were downregulated in ESCC tissues compared with normal tissues detected by microarray analysis and qRT-PCR.

**Figure S3:** linc00460 expression in different status from GEO Profiles. A.linc00460 expression is significantly lower in Neural tube defect patient than normal (GDS2470); B.linc00460 expression is decreased when knockdown LSD1 in neuroblastoma cell lines (GDS5281); C.MTX-sensitive HT29 colon adenocarcinoma cell line present higher linc00460 expression than MTX-resistant HT29 colon adenocarcinoma cell line (GDS3330).

**Figure S4:** Linc00460 expression of some other cancer cell lines of digestive system detected by qRT-PCR. GC: gastric cancer, including SGC-7901, AGS, MKN-45; CC: colon cancer, including HCT, MT29, LoVo; HCC: hepatocellular carcinoma, including HepG2, MHCC97h, SMMC7721, PLC; PC: pancreatic cancer, including BxPC-3, PANC-1.

**Table S1:** Sequences of qRT-PCR primers

**Table S2:** siRNA sequences

## Abbreviations

CCK-8: Cell Counting Kit-8
ChIP: chromatin immunoprecipitation
ESCC: esophageal squamous cell carcinoma
HOTAIR: HOX transcript antisense intergenic RNA
LncRNA: long noncoding RNA
