# Supplementary material for: A novel long noncoding RNA linc00460 up-regulated by CBP/P300 promotes carcinogenesis in esophageal squamous cell carcinoma
Source: Biosci Rep. 2017 Oct 17;37(5):BSR20171019. doi: 10.1042/BSR20171019 (PMC5964888; doi:10.1042/BSR20171019)
Supplement: Supplementary file 1 [file BSR20171019_Supp1.pdf]

Supplementary Figure S1

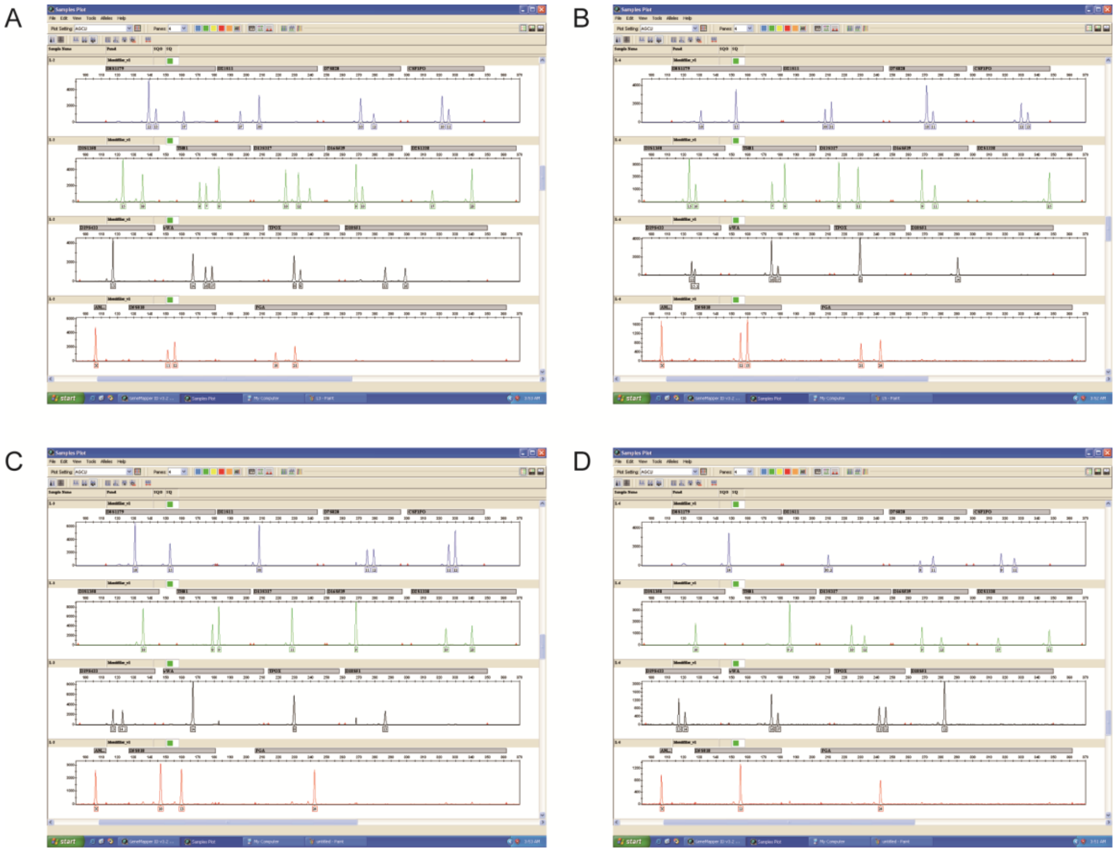

Figure S1: The results of cell line STR genotype. A, B, C, D represents for EC109, KYSE150, KYSE450 and Het-1A respectively.

Supplementary Figure S2

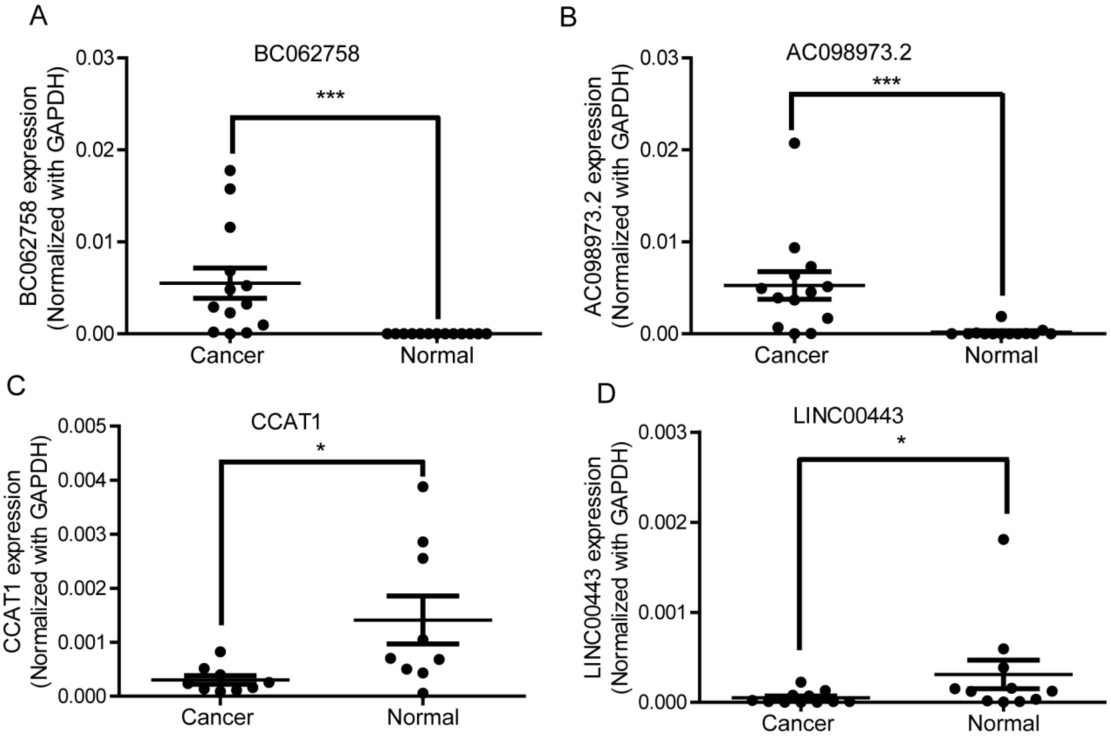


Figure S2: qRT-PCR confirmed the results of microarray. (A, B) LncRNA BC062758 and AC098973.2 were upregulated in ESCC tissues compared with normal tissues detected by microarray analysis and qRT-PCR; (C. D) LncRNA CCAT1 and LINC00443 were downregulated in ESCC tissues compared with normal tissues detected by microarray analysis and qRT-PCR.

Supplementary Figure S3

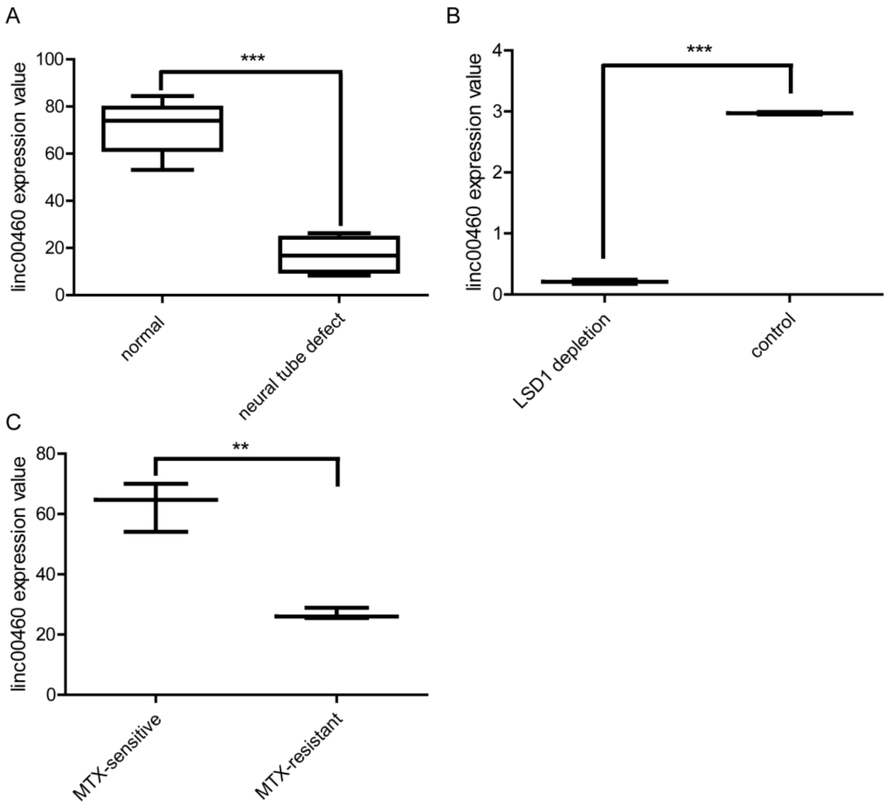


Figure S3. linc00460 expression in different status from GEO Profiles.

A.linc00460 expression is significantly lower in Neural tube defect patient than normal (GDS2470);

B.linc00460 expression is decreased when knockdown LSD1 in neuroblastoma cell lines (GDS5281);

C.MTX-sensitive HT29 colon adenocarcinoma cell line present higher linc00460 expression than MTX-resistant HT29 colon adenocarcinoma cell line (GDS3330).

Supplementary Figure S4

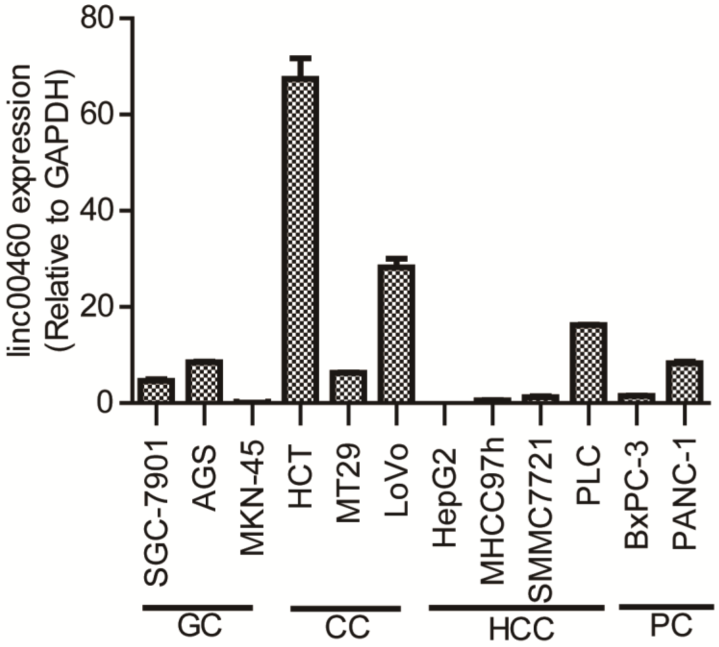


Figure S4: Linc00460 expression of some other cancer cell lines of digestive system detected by qRT-PCR.

GC: gastric cancer, including SGC-7901, AGS, MKN-45;

CC: colon cancer, including HCT, MT29, LoVo;

HCC: hepatocellular carcinoma, including HepG2, MHCC97h, SMMC7721, PLC;

PC: pancreatic cancer, including BxPC-3, PANC-1.

Supplementary Table S1

Table S1: Sequences of qRT-PCR primers

| qRT-PCR primers | Sequences 5’-3’ | PCR product length |
|---|---|---|
| GAPDH-F | GGGAGCCAAAAGGGTCATCA | 203bp |
| GAPDH-R | TGATGGCATGGACTGTGGTC | |
| Linc00460-F | GGGGACCGAGACCTATGAGA | 179bp |
| Linc00460-R | GAAAGCTGCAACATGCTCCC | |
| CBP-F | GTGCTGGCTGAGACCCTAAC | 125bp |
| CBP-R | GGCTGTCCAAATGGACTTGT | |
| P300-F | CAATGAGATCCAAGGGGAGA | 151bp |
| P300-R | ATGCATCTTTCTTCCGCACT | |
| **CHIP-qPCR primer** | **Sequences 5’-3’** | **PCR product length** |
| Linc00460-F | GGGGACTCATCTCCTCAAACC | 74bp |
| Linc00460-R | CATGGCACTTCCGTCACCTC | |

Supplementary Table S2

Table S2: siRNA sequences

| linc00460 siRNAs | Sequences 5’-3’ | siRNA length |
| --- | --- | --- |
| si-linc00460-1 | CUCCA GCCCU GUUAG AAAUTT | 21nt |
| si-linc00460-2 | GGUAC CCAGA CAUUG UUAUTT | 21nt |
| si-linc00460-3 | CCAGA UAAGU GCCCG AAUATT | 21nt |
| CBP siRNAs | Sequences 5’-3’ | siRNA length |
| si-CBP-1 | CGGCA CAGCC TCTCA GTCA | 19nt |
| si-CBP-2 | GGAGC CATCT AGTGC ATAA | 19nt |
| si-CBP-3 | GGAAC TAGAA CAAGA AGAA | 19nt |
| P300 siRNAs | Sequences 5’-3’ | siRNA length |
| si-P300-1 | GCACG AACTA GGAAA GAAA | 19nt |
| si-P300-2 | CGACT TACCA GATGA ATTA | 19nt |
| si-P300-3 | GCACA AATGT CTAGT TCTT | 19nt |
